# With Some Help From My Network: Supplementing eHealth Literacy With Social Ties

**DOI:** 10.2196/jmir.6472

**Published:** 2017-03-30

**Authors:** Tsahi (Zack) Hayat, Esther Brainin, Efrat Neter

**Affiliations:** ^1^ Interdisciplinary Center Herzliya Israel; ^2^ Ruppin Academic Center Emek Hefer Israel

**Keywords:** eHealth literacy, consumer health information, outcomes assessment, ethnicity

## Abstract

**Background:**

eHealth literacy is defined as the ability to seek, find, understand, and appraise health information from electronic sources and apply knowledge gained to addressing or solving a health problem. Previous research has shown high reliance on both online and face-to-face interpersonal sources when sharing and receiving health information.

**Objective:**

In this paper, we examine these interpersonal sources and their interplay with respondents’ eHealth literacy and perceived health outcomes. Specifically, we look at how the relationship between eHealth literacy and health outcomes is moderated by (1) finding help while performing online activities, (2) finding others with similar health concerns online, and (3) the importance of finding others with similar health concerns for people from ethnic minorities, specifically Palestinian citizens of Israel versus Israeli Jews.

**Methods:**

We used a nationally representative random-digit dial telephone household survey of an Israeli adult population (age ≥21 years, N=819). The collected data were analyzed using two regression models. The first examined how the correlation between eHealth literacy and perceived outcomes was moderated by the availability of help. The second examined how the correlation between eHealth literacy and perceived outcomes was moderated by finding others with similar health concerns and by ethnicity.

**Results:**

Respondents with low eHealth literacy who were able to recruit help when performing online activities demonstrated higher perceived health outcomes compared to similar respondents who did not find help. Respondents with low eHealth literacy, who were able to find others with similar health concerns (online), demonstrated higher perceived health outcomes when compared to similar respondents who did not find others with similar health concerns. Finally, finding similar others online was more helpful in enhancing health outcomes for ethnic minorities; Palestinian citizens of Israel gained more health benefits by finding similar others compared to Israeli Jews.

**Conclusions:**

Although the availability of help and the notion of ethnicity have been discussed extensively within the context of social capital and health, our findings offer initial evidence for the relevancy of these concepts when studying individuals’ eHealth literacy. Specifically, our findings enable a better understanding of the role of social ties and ethnicity in moderating the interplay between eHealth literacy and perceived health outcomes. Given the increased importance of eHealth information, our findings enhance understanding of how social ties can potentially compensate for low eHealth literacy.

## Introduction

The term eHealth refers to “the use of emerging information and communications technology to improve or enable health and health care” [1]. In the rapidly developing use of the Internet in society, eHealth literacy has become an important prerequisite for promoting healthy behavior [2,3]. Previous research has shown high reliance on both online and face-to-face interpersonal sources in searching for relevant health information and on forming decisions regarding different health-related topics [4,5]. Within the Israeli context, it was shown that consulting with other online users on health-related topics led to increased levels of health information and increased usage levels of online health services [6,7].

Despite the central role of interpersonal communication in accessing health information, research investigating how interpersonal communication interplays with eHealth literacy is scarce. In this study, we examine the impact of social ties on eHealth literacy among ethnic groups in Israel. Specifically, we investigate to what extent the availability of offline help when searching for health information online and finding other online individuals with similar health conditions can supplement individuals’ low eHealth literacy, which in turn can help health consumers achieve positive outcomes when using the Internet for health purposes.

### Related Work

This section outlines the theoretical concepts used to frame the paper and provides a summary of past and present research. The conceptual framework offers a brief perspective on the association between eHealth literacy and perceived health outcomes. This section also introduces the concepts of interpersonal communication, social networks, and social capital.

#### eHealth Literacy

Health literacy is defined as the ability of an individual to obtain, process, and understand basic health information in order to make appropriate decisions concerning health [8,9], whereas eHealth literacy is defined as “the ability to seek, find, understand and appraise health information from electronic sources and apply knowledge gained to addressing or solving a health problem” [10]. According to this definition, eHealth literacy encompasses six types of literacy: traditional (literacy and numeracy), information, media, health, computer, and scientific. Of these, media and computer literacies are unique to the Internet context, with eHealth media literacy being the awareness of media bias or perspective, the ability to discern both explicit and implicit meaning from media messages, and to derive meaning from media messages. The literature includes other definitions of perceived media capability or efficacy, but these were not specific to health information on the Internet [11].

Having the composite skills of eHealth literacy allows health consumers to achieve positive outcomes from using the Internet for health purposes. eHealth literacy has the potential to both protect consumers from harm and empower them to fully participate in informed health-related decision making [10]. People with high levels of eHealth literacy are also more aware of the risk of encountering unreliable information on the Internet [7,12]. On the other hand, the extension of digital resources to the health domain in the form of eHealth literacy can also create new gaps between health consumers [11]. eHealth literacy hinges not on the mere access to technology, but rather on the skill to apply the accessed knowledge [13], thus lending support to the hypothesis that information technology is creating a new social inequality rather than leveling social discrepancies [14].

The importance of assessing eHealth literacy is highlighted by recent findings suggesting that instead of basing health care treatment decisions on professional weighing of risks and benefits for different treatments and outcomes, individual preferences regarding health treatments are based in reality on limited information influenced by prior individual and collective experience [15]. This can be partially understood by findings indicating that most interpersonal communication sources for health information are lay people, such as family and peers within one’s social network, rather than professional sources such as health care providers [16,17].

The examination of individuals’ social ties is gaining increased prominence in studies dealing with patients’ attainment of health information [18]. Specifically, more attention is being paid to the impact technologies such as email and the Internet have on the way individuals gather and share health information through their social ties [18]. Although these ties are important conduits for shared resources [18], much of the public health literature focuses either on patient-provider communication [19,20] or identifying and training people to serve as opinion leaders, peer leaders, or community health workers to aid in the implementation of designated health promotion/prevention disease interventions [21-23]. Less attention has been paid to identifying how similar health conditions are constructive in facilitating effective communication, for gaining access to information, for applying health information, and for increasing the benefits associated with such interpersonal health information sources [20]. More specifically, to the best of our knowledge, no attention has been paid in the interpersonal communication and health communication literature to study how social ties can potentially compensate for low eHealth literacy. Given the increased importance of social ties in attaining health information, and in light of the importance of eHealth literacy in understanding and appraising health information, we believe that by studying these two domains together we can better understand their joint potential contribution for enhancing health outcomes. Ways for bridging these two domains through combining emerging findings from the interpersonal ties literature with established literature in the health communication field are addressed in the next sections.

#### Social Ties

Social ties among individuals are often important conduits for sharing resources and can be described in such terms as their density, range, boundedness, and homogeneity [18]. Although social ties describe the structure of social interaction, social support describes the resources that are shared through these ties. Social support exists in a number of forms, including emotional support (eg, love, caring, and sympathy), instrumental support (eg, assistance with tangible needs), and informational support (eg, provision of advice or information) [24]. Social networks and social support can have a profound impact on health by improving health behaviors (eg, [25]) and allowing for the provision of health-conserving resources (eg, [26]).

Social ties are also a means for information exchange among individuals. For example, previous studies have shown that belonging to two or more community organizations is associated with increased knowledge of screening for colon cancer, and increased knowledge of levels of exercise and fruit and vegetable intake recommended to reduce cancer risk [24]. These results support the intuitive notion that the more social ties individuals have the more likely they are to be exposed to health-enhancing information. On the other hand, although social ties may encourage some to stop smoking [27], they may encourage others to take up the practice [27] or grow obese together [28].

As the previous examples indicate, the relationship between social ties and health communication are not as straightforward as they initially appear. One contentious issue involves the distinction between strong ties, defined as those that are frequent and multifaceted, and weak ties, defined as infrequent and unidimensional [29]. Conceptually, one might argue that strong ties are less likely to lead to the distribution of new information compared with weak ties because individuals tend to form stronger relationships with those who are more similar to themselves and thus strong ties may not be able to offer many innovative ideas in the course of a social exchange [29]. Thus, weak ties may be more important for exposing people to innovative ideas, particularly in terms of transferring health information [30]. Findings on this issue are mixed: one study from the United States found that people reported strong ties as the most important source of health information [31], whereas other research emphasized the importance of both strong and weak ties [32-34]. This finding was also documented in the Israeli context [6].

Social ties through which health information can be accessed and applied include contacts with professionals such as medical health providers and/or lay interpersonal communication sources, including other service consumers, family and friends, neighbors, and religious leaders [16,17]. Using the Internet to access health information, where it is widely prevalent, is steadily rising [7,35,36]. A recent study by Mesch [37], based on a representative sample, indicates a reliance on online communication for health purposes within the Israeli population both in accessing medical information as well as for communication on health-related topics. These findings on increased reliance on online sources of information among the general population are reiterated in other studies [32,38]. Due to the increasingly important role of computer-mediated communication (CMC) in the formation and preservation of social ties, as well as for information sharing, we focus in this paper on social ties facilitated by both CMC and face-to-face interactions, a field that has not been explored as a medium that can potentially supplement eHealth literacy.

#### Assistance Provision Through Social Capital

A concept that may further explain the role social ties play within the context of health communication is that of social capital [39-41]. There is no firm, unanimous definition of social capital, and the particular definition adopted by any given study is dependent on the discipline and level of investigation. In its simplest form, social capital can be defined as the social ties or connections through which one gains access to resources [42]. Coleman [43] defines social capital as a function of social structure producing advantage, whereas Bourdieu [42] defines social capital as “the aggregate of the actual or potential resources that are linked to possession of a durable network of more or less institutionalized relationships of mutual acquaintance and recognition” (p 248).

Despite the fact that social capital is a contentious and slippery term, the preceding definitions emphasize the notion that social capital resides not within the individual but rather in the relationships that an individual or group has with others. For the purpose of this study, social capital theory is defined as the sum of the actual and potential resources embedded within, available through, and derived from an individual’s network of relationships. Social capital comprises both the network and the assets that may be mobilized through it [44]. Such value can also be associated and facilitate the flow of information [45].

The importance of these social capital within the context of health is seminal, given that such resources has been found to play a central role in enhancing health [46,47]. Previous work have shown that social capital has a positive impact on participants’ emotional and physical well-being, either through gratifying the health information needs of individuals or through communication with health care providers and with other lay people [46,47]. Although the effect of social capital on health has been extensively studied in the past three decades [48], relatively little work has been done on the role social ties plays for individuals with low eHealth literacy. Such individuals can potentially access resources in their social networks (ie, other individuals who can guide them when searching for health-related information online); such assistance can supplement their low eHealth literacy and enhance their perceived health outcomes. This study will not measure the social capital of individuals, although it is guided by the social capital literature in studying the benefits social ties can offer for individuals’ perceived health outcomes. Specifically, in light of this literature, we are interested in examining whether the services and resources, available through the social ties of individuals, can help individuals to seek, find, understand, and appraise health information from electronic sources. Thus, our first hypothesis is that at low levels of eHealth literacy, finding others who can help perform online activities is associated with higher perceived health outcomes, whereas at high levels of eHealth literacy, perceived health outcomes do not vary as a function of finding others who can help.

#### Information Provision Through Similar Others

Interpersonal communications are central to social capital [20]. Interpersonal communication is the medium through which individuals and groups create, foster, alter, and terminate their social ties. Expressing needs and negotiating assistance constitute key aspects of social support that draw on interpersonal communications. Through the iterative communication between individuals, societies collectively create (or fail to create) the social participation, norms of reciprocity, and group trust that are the hallmarks of social capital. Without interpersonal communication, these social processes would lose their meaning and indeed cease to exist [20].

Despite the tremendous amount of information available on the Web, research has shown that users continue to rely on people in their networks when seeking various types of information. Such work has examined different domains of information search, ranging from recreational activities [49] to cultural content [50], and has found that users supplement online sources with advice they obtain from friends and family. Furthermore, research on information flow and attitudes within social networks suggests that ties between individuals and similar others can promote the exchange of relevant information among peers [51] and affect their attitudes toward that information [52,53]. These findings are attributed to the fact that most people tend to rely on the subjective evaluation of friends, family, and trusted others rather than on scientific evidence to form an opinion and make a decision about something [18]. Moreover, individuals are more likely to be receptive to information shared by others who are similar to them [54]. Although recent studies have documented the tendency of Internet users to seek others who might share the same health concerns they have [5], studies thus far have not explicitly addressed the role of information received from similar others as a mechanism that can compensate for low eHealth literacy. Previous studies that looked at interpersonal health communication (eg, [55]) have shown that such communication tends to use lay terminology and plain language, thus ‘‘translating’’ complicated health information for one another. Additionally, these interactions provide opportunities to hear about the personal experiences of other network members, who are perceived as both dealing with similar concerns and as unbiasedly sharing relevant information [56,57]. Thus, our second hypothesis is that at low levels of eHealth literacy, finding others with similar health concerns is associated with higher perceived health outcomes, whereas at high levels of eHealth literacy, perceived health outcomes do not vary as a function of finding others with similar health concerns.

#### Information Provision Through Similar Others and Ethnicity

The findings regarding the association between social ties and health outcomes have been inconsistent. Some studies have found a beneficial effect of social ties over health outcomes both internationally [58] and in Israel [6,37], whereas others have demonstrated either null or negative associations [59]. These inconsistencies may reflect the reality that social ties can be both an asset and a liability for health. Social ties are believed to benefit health through access to resources such as emotional and material support and health information generated by social networks [60]. Detrimental health effects of social ties may occur because of unmanageable demands of networks or exposure to unhealthy behaviors such as smoking [61]. Social ties may provide different positive and negative resources, and there is some evidence that the potential benefits of different types of social networks for health may vary, for instance by social classes [48,62]. Among ethnic minorities, local social ties might exclude people from access to health information. Furthermore, mechanisms of control and social pressure, resulting from these local social ties, can cause social exclusion [62] and lead to further deteriorations in health.

In Israel, disparities in health exist between the three main population groups: nonimmigrant Jews, immigrants from the former Soviet Union (arriving in Israel since 1990), and Palestinian citizens of Israel (PCI) [37,63]. Specifically, inequalities in the use of health care services based on ethnicity are also evident: PCI are more likely to visit a general practitioner, less likely to visit a specialist, and more likely to be hospitalized compared to Jewish Israelis [64]. These differences cannot be attributed to insurance factors because all Israeli residents have access to universal health care coverage. However, several structural barriers may explain differences in access to services. Although primary services are available in Arab localities (villages and midsize towns), specialist clinics are more likely to be located in large cities. Thus, PCI must overcome both language and geographic barriers to access specialists: sometimes they have to travel an hour each way to Jewish cities where specialized clinics are located, missing work and incurring transportation costs [65]. These challenges may also explain why PCI are more likely than the ethnic majority to access health information online because such information is accessible in Arabic and it can potentially eliminate transportation costs [37].

Barriers in communication with health care specialists have been documented in low-literate and minority communities; these individuals are more likely to seek advice from friends and family than from trained health care providers or peer-reviewed journals [65]. It is not surprising that studies both in Israel [6] and the United States [66,67] documented that members of ethnic minorities are likely to rely more on their social ties as sources for health information compared to their ethnic majority counterparts, who tend to rely more on mass media sources.

Nevertheless, the benefits of advice from friends and family might not be available or beneficial to everyone in the same way. For instance, social ties might benefit those who are better off in society, but constrain or even exclude people with a lower socioeconomic status or in a minority position [62]. A recent study offered a systematic review of 60 studies examining the interactions between the benefits associated with social ties and socioeconomic-disadvantaged groups [48]. The article reported findings indicating a greater health benefit of social ties for people with a disadvantaged position in society, and no effects or limited health benefits for those with a position higher up the social ladder. People with high perceived availability of social ties can turn to these social ties, and use the resources available through these ties to enjoy greater perceived health, than expected considering their low socioeconomic status [48]. We hypothesize that similar findings will be evident when studying perceived health outcomes among PCI. Thus, our third hypothesis is that the interaction between eHealth literacy, finding others with similar health concerns, and perceived outcomes is stronger for PCI compared to Jewish Israelis.

## Methods

### Data Collection and Sample Characteristics

Data were collected in a nationally representative random-digital dial (RDD) telephone household survey of Israeli adult population (aged 21 years and older) conducted in November 2014 (landlines and mobile combined). The following RDD-based sampling procedure was used: statistical areas were divided into four strata layers according to (1) population group (Jews, PCI, and mixed localities), (2) seven geographical districts, (3) size of settlements (large cities, small towns and villages), and (4) the locality’s socioeconomic status index. These strata were based on the Israeli Central Bureau of Statistics classification. Sampling employed a dual-frame design, incorporating two selection stages. The first frame was designed to provide national coverage of the eligible population and was guided by the statistical areas defined by the Israeli Central Bureau of Statistics; the second frame sampled households within each statistical area. Calls were placed to 1789 residential households; 1628 eligible potential respondents were identified (ineligible numbers included eight fax numbers and 153 disconnected phones), of whom 819 agreed to be interviewed, representing a 50.31% response rate. The interviews were conducted in Hebrew, Russian, or Arabic by professional interviewers who underwent a special training session to familiarize them with the questionnaire’s terminology. The interviewers also read the interviewees the consent form (adapted from [12]) and invited the interviewee to ask any questions she or he might have. The interviewers conducted the telephone survey using computer-assisted telephone interviewing software.

### Measures

Perceived health outcomes of seeking health information on the Internet, the dependent variable in our study, was measured using the item “Do you agree or disagree that seeking health information on the Internet...?” followed by a list of nine outcomes (adapted from [4,68,69]). Responses were expressed on a five-point scale from 1 (strongly agree) to 5 (strongly disagree). The respondents also had the option to indicate as a response to each item that they either “don’t know” or that the question is “irrelevant.” For each respondent, a total mean score of perceived health-related outcomes was computed (alpha=.87, mean 1.48, SD 1.64) (see Multimedia Appendix 1 for a full list of the items used in the survey).

The independent variables in this study included perceived ease of obtaining face-to-face help in performing online activities, eHealth literacy, finding others online with similar health concerns, and ethnicity. Each of these variables were measured using the following items. Ease of obtaining help in performing online activities was assessed by responses to the question “When you need advice or help surfing the Internet, for example help in finding a particular site or service, how easy is it for you to find someone who will help you?” on a five-point Likert scale ranging from 1 (very difficult) to 5 (very easy) [70] (mean 1.94, SD 2.12). This item assessed the help attained through face-to-face interactions. Perceived eHealth literacy was assessed using the eHealth Literacy Scale (eHEALS) [10], a scale consisting of eight items on a five-point Likert scale ranging from 1 (strongly disagree) to 5 (strongly agree). The scale was previously translated to Hebrew, Arabic, and Russian [7] (alpha=.78, mean 1.64, SD 1.77). Finding others with similar health concerns was assessed by responses to the question “During the past 12 months, how often have you used the Internet for finding others with health issues or concerns similar to the ones you are facing?” on a five-point Likert scale ranging from 1 (very rarely) to 5 (very often) [5] (mean 0.75, SD 0.84). This item assessed only the online ties with similar others. Ethnicity was measured by self-identification (Jewish / Arab Muslim / Arab Christian / Arab Druze).

Our control variables were Internet activities (Web 1.0 and Web 2.0) [71] assessed by reported frequency of 15 digital activities on a five-point Likert scale from 1 (never) to 5 (very often). The Web 1.0 activity index was assessed using a scale consisting of eight items (eg, searches for driving instructions, searches for a product/service) (alpha=.81, mean 2.38, SD 0.71). The Web 2.0 activity index was assessed using a scale consisting of seven items (eg, manages a personal site, uploads photos/video or responds to photos by others) (alpha=.83, mean 1.59, SD 0.71) [72]. Sociodemographic information on age, gender, and education were obtained as part of the background variables. Sociodemographic information, specifically age and education, are known to be strong predictors of online and offline health information behavior [7] and needed to be controlled in our analysis.

### Data Analysis

We employed two linear regression models to study the interplay between eHealth literacy and perceived outcomes of seeking health information on the Internet. The first regression model, a three-step hierarchical multiple regression model, assessed the interaction effect of help availability and eHealth literacy on the perceived health outcomes of information search. Our second regression model, a four-step hierarchical multiple regression model, assessed the interaction effect of finding others with similar health concerns and eHealth literacy on the perceived health outcomes of an individual. The second model also assessed a three-way interaction between finding others with similar health problems, eHealth literacy, and ethnicity on the perceived health outcomes of an individual, for PCI, and for Jewish Israelis.

For both regression models, the Durbin-Watson statistic was used to investigate the assumption of independence. Normal probability plots were used to investigate the normality of error terms and homoscedasticity was tested by observing the scatterplot of the residuals and the predicted value. These checks identified no violations of multiple regression assumptions. All statistical tests were one-tailed and a significance level of *P*<.001 was set for all analyses.

To facilitate the interpretation of the interactions, all continuous variables used in our models were standardized [73]. To calculate the statistical power of this study to reject false null hypotheses, we conducted a post hoc statistical power test [74,75]. With 11 predictors in the regression analysis, an observed *R*^2^ of .88, a sample size of 487, and alpha=.05, the test results indicated an observed power of 1.0.

## Results

Among the Israeli Jew respondents, the mean age was 51.1 (SD 17.2) years, and 549 of 683 (80.4%) had Internet access. Among the PCI respondents, the mean age was 42.5 (SD 13.6) years, and 112 of 136 (82.4%) had Internet access. Table 1 provides the gender and education distribution, respectively, within our sample. Furthermore, given that this study focused on individuals who use the Internet for acquiring health information, it is important to note that 49.1% of our sample indicated that they use the Internet as a source for health information. This figure is consistent with current studies conducted in the Israeli context [37].

**Table 1:** Demographic distribution among the respondents (N=819).

Demographics		Israeli Jews (n=683)	PCI (n=136)
Age (years), mean (SD)		51.1 (17.2)	42.5 (13.6)
**Gender, n (%)**			
	Male	328 (48.0)	67 (49.3)
	Female	355 (51.9)	69 (50.7)
**Education, n (%)**			
	High school or less	303 (44.2)	66 (48.6)
	Professional degree	125 (18.3)	16 (11.8)
	Partial academic degree	19 (2.8)	7 (5.1)
	Bachelor’s degree	147 (21.5)	35 (25.7)
	Master’s degree or above	89 (13.0)	12 (8.8)

A three-step hierarchical multiple regression model was conducted to examine our first hypothesis, which stated that the association between eHealth literacy and perceived outcomes is moderated by availability of help (see Multimedia Appendix 2 for the distribution of the following variables: ease of obtaining help in performing online activities, perceived eHealth literacy, finding others with similar health concerns, and the outcome measures).

**Figure 1:**
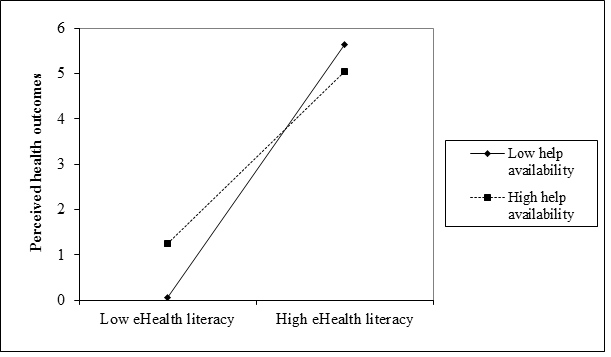
Interaction effect of help availability and eHealth literacy on the perceived health outcomes of information search (n=487).

As can be seen in Table 2, age, gender, education, and Internet activity were entered in the first step. eHealth literacy and help availability were entered in the second step and their interaction term in the third step. The overall model was significant (step 3: Δ *R*^2^=.009; *F*_7,479_=352.22, *P*<.001). To test the appropriateness of our steps, we assessed the *R*^2^ increase in step 2 relative to step 1, as well as for step 3 relative to step 2, with an *F* test. The results of the *F* test show that the respective *F* changes of step 2 and step 3 were 1042.56 (*P*<.001) and 27.81 (*P*<.001), respectively. Regression coefficients and significance values are presented in Table 2. As indicated by our findings, there was a significant main effect for eHealth literacy (beta=2.35, SE=0.31; *t*_480_=46.29, *P*<.001), a significant main effect for help availability (beta=0.15, SE=0.30; *t*_480_=3.17, *P*=.003), and a significant interaction (beta=–0.45, SE=0.51; *t*_479_=–5.27, *P*<.001). The interaction plot, depicted in Figure 1, suggests that high help availability yielded higher perceived outcomes when eHealth literacy was low, as compared to lower perceived outcomes when there was low help availability. Simple slopes tests, following Cohen et al [76], were conducted at one standard deviation above and below the mean of help availability. Both slopes were significant (*P*<.001). Note that the background variables, which significantly predicted the perceived outcomes in the first step, did not contribute to the prediction of perceived outcomes once eHealth literacy and the availability of help were included as predictors. Gender was not associated with perceived outcomes even in the first step. Thus, our first hypothesis was supported.

**Table 2:** Standardized variables included in the hierarchical regression model predicting perceived outcomes.

Independent variables	Step 1 (n=508)			Step 2 (n=487)				Step 3 (n=487)		
	β^a^	*t*_507_	*P*	β^a^	*t*_486_	*P*	β^a^		*t*_486_	*P*
Age	–2.59	–3.69	<.001	0.02	0.07	.23	0.12		0.39	.22
Gender	0.64	0.95	.08	–0.28	–0.95	.13	–0.30		–1.03	.10
Education	2.81	3.68	<.001	0.09	0.27	.55	0.14		0.43	.75
Internet activity	1.82	2.57	.006	–0.60	–1.92	.09	–0.44		–1.45	.10
eHealth literacy				4.21	44.81	<.001	2.35		46.29	<.001
Availability of help				0.21	3.52	<.001	0.15		3.17	.004
eHealth literacy × availability of help							–0.45		–5.27	<.001

^a^ Because all continuous variables were standardized, betas for continuous predictors correspond to standardized regression coefficients.

**Figure 2:**
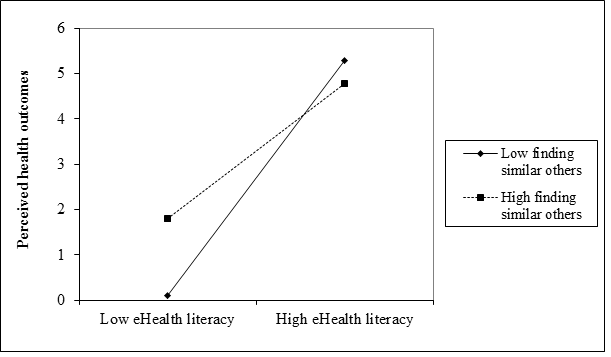
Interaction effect of finding others with similar health concerns and eHealth literacy on the perceived health outcomes of an individual (n=493).

A four-step hierarchical multiple regression model was used to examine our second and third hypotheses. Our second hypothesis stated that the association between eHealth literacy and perceived outcomes is moderated by finding others with similar health concerns, whereas our third hypothesis posited that this interaction between eHealth literacy and finding others with similar health concerns on perceived outcomes is stronger for PCI (compared to Jewish Israelis). As can be seen in Table 3, age, gender, education, and Internet activity were entered in the first step. eHealth literacy, finding others with similar health concerns, and ethnicity (as a dummy variable; Israeli Jews were coded as 0), were entered in the second step, their interaction terms in the third step, and the three-way interaction (eHealth literacy × finding others with similar health concerns × ethnicity) was entered in the fourth step. The overall model was significant (step 4: Δ *R*^2^=.02; *F*_7,485_=377.97, *P*<.001). To test the appropriateness of our steps, we assess the *R*^2^ increase in step 2 relative to step 1, for step 3 relative to step 2, as well as for step 4 relative to step 3 with an *F* test. The results of the *F* test show that the respective *F* changes of step 2, step 3, and step 4 were 1030.14 (*P*<.001), 64.29 (*P*<.001), and 58.34 (*P*<.001). Regression coefficients and significances are presented in Table 3. Our findings (see step 3 in Table 3) indicated a significant main effect for eHealth literacy (beta=2.04, SE=0.50; *t*_486_=24.99, *P*<.001), a significant main effect for finding others with similar health concerns (beta=.30, SE=0.48; *t*_486_=0.87, *P*<.001), and a significant interaction (beta=–0.55, SE=0.53; *t*_485_=–0.23, *P*<.001). The interaction plot, depicted in Figure 2, suggests that finding others with similar health concerns yielded higher perceived outcomes when eHealth literacy was low, as compared to lower perceived outcomes when the rate of finding others with similar health concerns was low. Simple slopes tests at one standard deviation above and below the mean of finding others with similar health concerns were conducted. Both slopes were significant (*P*<.001). Thus, our second hypothesis was supported.

**Table 3:** Standardized variables included in the hierarchical regression model predicting perceived outcomes.

Independent variables	Step 1 (n=508)			Step 2 (n=493)			Step 3 (n=493)			Step 4 (n=493)		
	β^a^	*t*_507_	*P*	β^a^	*t*_492_	*P*	β^a^	*t*_492_	*P*	β^a^	*t*_492_	*P*
Age	–2.61	–3.73	<.001	0.19	0.61	.30	0.24	0.80	.31	0.24	0.60	.31
Gender (0=male)	0.65	0.95	.08	–0.08	–0.25	.15	–0.06	–0.20	.17	–0.08	–0.32	.19
Education	2.80	3.68	<.001	–0.05	–0.14	.37	0.11	0.35	.21	0.08	0.28	.20
Internet activity	1.87	2.66	.006	–0.48	–0.15	.07	–0.19	–0.64	.08	–0.27	0.57	.09
eHealth literacy				2.78	25.86	<.001	2.04	24.99	<.001	1.78	21.42	<.001
Finding others with similar health concerns				0.21	0.68	.008	0.3	0.87	<.001	0.28	0.85	<.001
Ethnicity (0=Jewish Israelis)				–0.24	–0.18	.004	–0.17	–0.22	.003	–0.18	0.25	.007
eHealth literacy × finding others with similar health concerns							–0.55	–0.23	<.001	–0.48	–0.31	<.001
eHealth literacy × ethnicity							–0.23	0.63	.007	–0.16	0.41	.95
Ethnicity × finding others with similar health concerns							–0.41	0.28	.004	–0.32	0.38	.62
eHealth literacy × finding others with similar health concerns × ethnicity										–0.54	–0.72	.008

^a^ Because all continuous variables were standardized, betas for continuous predictors correspond to standardized regression coefficients.

**Figure 3:**
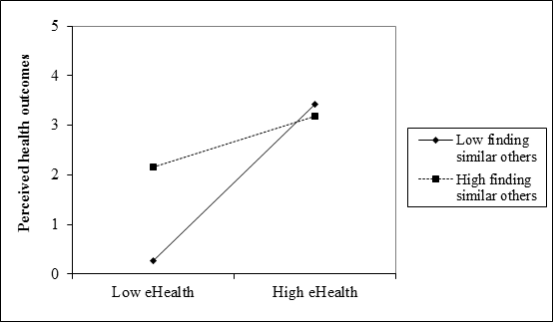
Three-way interaction effect of finding others with similar health problems, eHealth literacy, and ethnicity with the perceived health outcomes for PCI.

**Figure 4:**
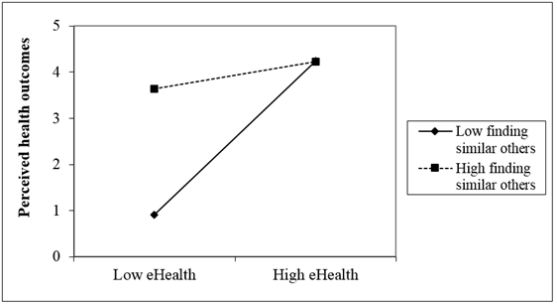
Three-way interaction effect of finding others with similar health problems, eHealth literacy, and ethnicity with the perceived health outcomes for Jewish Israelis.

As depicted in Table 3, step 4 of our analysis revealed a significant three-way interaction (finding similar others × eHealth literacy × ethnicity) for perceived health outcomes (β=–0.54, SE=0.51; *t*_482_=–0.72, *P*=.003). This significant three-way interaction is depicted in Figure 3 and Figure 4 indicating that the interaction between eHealth literacy and finding others with similar problems is weaker for Jewish Israelis (β=–0.53; *t*_482_=–0.64, *P*=.02) than for PCI (β=0.69, *t*_482_=–0.75, *P*=.03). In either case, finding others with similar health concerns yielded higher perceived outcomes when eHealth literacy was low as compared to lower perceived outcomes when the rates of findings others with similar health concerns was lower. This pattern diminishes under higher levels of eHealth literacy. We conducted simple slopes tests, separately for PCI and Jewish Israelis, at one standard deviation above and below the mean of finding similar others. Both slopes were significant for Jewish Israelis (*P*=.03) and for PCI (*P*=.02). Thus, our third hypothesis was supported.

## Discussion

### Main Findings

The contribution of this paper to the field of eHealth literacy is threefold. First, we showed how the availability of help when searching for health information online can enhance the perceived health outcomes of individuals with low eHealth literacy. We then showed that finding others with similar health concerns online can also enhance the perceived health outcomes of individuals with low eHealth literacy. Finally, we showed that finding similar others online is more helpful in enhancing perceived health outcomes for ethnic minorities; in our case, PCI gained more by finding similar others when compared with Israeli Jews. Although the availability of help and the notion of ethnicity have been discussed extensively within the context of social capital and health, our findings offer initial evidence for the relevance of these concepts for eHealth literacy as well. It is important to note that the differences in perceived health outcomes are very large between people with high and low eHealth literacy (despite the influence of help available and the ability to find others with the same health problems) as depicted by the main effect of eHealth literacy on perceived health outcomes (see Tables 2 and 3). Thus, availability of help and finding similar others can elevate individuals with low eHealth literacy, and increase their perceived health outcomes, but not to eradicate the gap between people with low and high eHealth literacy.

### Comparison With Prior Work

Previous studies have stressed the notion that some Internet usage activities are more beneficial or advantageous for Internet users than others. Some activities offer users more chances and resources in moving forward in their career, work, education, and societal position than others that are mainly consumptive or entertaining [77]. People’s use of the Internet as an important source for making health-related decisions is an indication of their having adopted an “Internet-oriented lifestyle,” which leaves different “footprints” on the users’ lives, manifested in an increase of social and financial returns [77]. Within the context of health, previous studies have also highlighted the importance of enhancing eHealth literacy to improve individuals’ perceived health outcomes [10].

The vast majority of attention in eHealth literacy research has been focused on information accessibility, namely the delivery and readability of health-related information [78]. Accessible information that one understands is a necessary but not sufficient condition for addressing eHealth literacy. One’s ability to apply the information in making health care decisions based on the information accessed is also an important part of eHealth literacy. Yet, even if one assumes that information is written at a reading level that can be understood by its readers, translating it into a culturally appropriate vernacular and delivering it via a communication channel that is accepted and easily accessed does not guarantee that the information will be utilized as it was intended [79]. At best, having accurate information will lead to a basic understanding of what the message sender desires the recipient both to know and to do, which is what Nutbeam characterized as functional health literacy [80]. Functional health literacy is a vital first step to realizing improvements in many health-related outcomes. Failing to move beyond functional health literacy can be likened to a health care system that is only concerned with emergency medicine, rather than addressing problems at their source. Nutbeam [80] has gone on to describe a second level of health literacy; namely, interactive health literacy. This level refers to the personal capacity to build skills and “act independently” (p 266 [80]) when armed with factual information. Finally, critical health literacy is the empowerment of an individual to promote his or her own health outcomes despite difficult economic or social situations.

Addressing the interactive and critical eHealth literacy needs of a population presents an increasingly complex set of issues for health providers and researchers. Similar to health promotion efforts, the scope of the problem becomes exponentially large when one considers the need to equip and empower people to be their own educated, capable health promoters. Today’s researchers and health care providers can benefit from the current proliferation of eHealth literacy research. Some researchers have begun to consider how such large-scale efforts could be conceptualized. For example, Ratzan [81] suggested a four-pronged approach, including integrated marketing communication (drawing on public relations and social marketing experts), health education (using the Internet and other multimedia channels), shared decision making (building partnerships with key groups), and efforts to increase the social capital and social ties of disenfranchised groups (providing social and relationship resources that are more scarce for underprivileged people). Kickbusch [82] has suggested that by considering health literacy broadly (as opposed to isolating diseases or specific health risks) and working to increase a population’s social capital, health care advocates can achieve a more integrated and sustained program of health and social change. Simply put, increasing a population’s health literacy across multiple health contexts will result in that population being empowered to take more control when addressing future health-related challenges.

In an attempt to expand this eHealth literacy agenda, our findings enable a better understanding of the role social ties and ethnicity play in moderating the interplay between eHealth literacy and perceived health outcomes. Specifically, we highlight the potential benefit of social ties in compensating for low eHealth literacy. In order to achieve this goal, this paper utilized the social capital and eHealth literacy frameworks for studying the role social ties play within the process of accessing and utilizing health information. As our findings indicate, social ties can enhance both the interactive and critical eHealth literacy needs (as evident from their importance in moderating the association between eHealth literacy and perceived health outcomes). Thus, we suggest that both researchers and practitioners will incorporate social ties into the study and implementation of eHealth literacy enhancement. Although many large-scale efforts to enhance eHealth literacy implicitly discuss the importance of social ties and social capital within the context of enhancing eHealth literacy, our findings provide a clear indication for the potential role social ties can play within this context.

Our work joins the extensive evidence indicating that social ties have implications in terms of both exposure to diverse information and the ability to utilize it [32,83]. In this work, we add to the literature on social ties and social capital by gaining a better understanding of the mechanisms through which social ties can moderate the interplay between eHealth literacy and perceived health outcome. We join previous work that highlight the notion that social capital is not based solely on face-to-face relationships, but also on online relationships (eg, [84]). Such a conceptualization allows us to investigate whether and to what extent people who encounter obstacles in obtaining health information through the Internet turn to their social ties for help and how these ties benefit people from different ethnic backgrounds. The fundamental notion of this study is that people who encounter barriers in obtaining health information and services through the Internet turn to their social ties for help [1]. The contribution of social ties in the context of eHealth is less widely acknowledged and this study addresses this gap.

### Limitations

Our findings are hampered by three major limitations. First, the cross-sectional design of the study precludes causal conclusions and allows us to draw conclusions regarding only correlated relationships. For example, we can assume only that finding others with similar health concerns online mediates the interplay between eHealth literacy and perceived health outcomes, not that it affects eHealth literacy. Second, we did not measure performed eHealth literacy and health outcomes, but rather perceived efficacy of searching and using health information on the Internet. Although previous studies have found an association between perceived health information (eg, control of illness and perceived understanding of the illness) and actual health outcomes (eg, [85]), future studies should use measures of performed eHealth literacy and health outcomes. Indeed, measures for performed digital literacy [86] and measures for health literacy [87] exist. These measures may serve as inspiration for a measure that captures performed eHealth literacy and health outcomes. Future studies, possibly based on “big data” that records and monitors actual activities, may shed more light on the association between online usage and gains, at least measuring the usage more accurately. Thirdly, although this study points to the potential importance of social ties in moderating the association between eHealth literacy and perceived health outcomes, future studies could explore the extent to which people are indeed able to turn to their social ties, ask others for help or find online peers, and whether the interplay of eHealth literacy and social ties is more important for certain types of health information seeking (eg, whether this interplay is more important for enhancing information about prevention, symptom identification and self-diagnosis, or for learning about potential treatments). Finally, in this study, we did not look at the specific features of individuals’ social ties (eg, strength and diversity of social ties) and how such features might affect eHealth literacy. More specifically, no attention was directed to an exploration of the types of social ties (eg, family, local friends, neighbors, online contacts) that are more effective in compensating for low eHealth literacy. Given the documented importance of interpersonal ties in the attainment of health information [16,17], we suggest that future studies should address this theoretical and empirical gap.

### Conclusions

One of the most powerful trends is the increasing penetration of new CMC and the role they play in health. This paper suggests an additional contribution CMC offers: potential enhancement of eHealth literacy through interpersonal sources of health information. This study is one of the first empirical works that systematically investigates the role of interpersonal ties in eHealth literacy. More specifically, previous studies in the interpersonal communication and health communication literature have not identified how the availability of other people while searching for health information, and their attributes, may promote perceived health outcomes. In this paper, we address this theoretical and empirical gap by combining the literature on social ties with established literature in the health communication field. By doing so, we were able to offer a new perspective on the role of social ties in compensating for low eHealth literacy and in increasing perceived health outcomes.

Although this study is anchored in the Israeli context, given that ethnic minorities in other countries rely on interpersonal sources for attaining health information (eg, [18]), and the evident increase among Internet users in turning to find others who might share their health concerns (eg, [5]), we believe that our findings are also relevant for other cultural contexts.

## Appendix

Multimedia Appendix 1 Items used for assessing respondents’ perceived health outcomes from using the Internet for health purposes.

Multimedia Appendix 2 Descriptive statistics of the ease of obtaining help in performing online activities, perceived eHealth literacy, finding others with similar health concerns, and the perceived outcome of Internet use measures.

## Abbreviations

CMC: computer-mediated communication
PCI: Palestinian citizens of Israel
RDD: random-digital dial
