# Supplementary material for: With Some Help From My Network: Supplementing eHealth Literacy With Social Ties
Source: J Med Internet Res. 2017 Mar 30;19(3):e98. doi: 10.2196/jmir.6472 (PMC5391437; doi:10.2196/jmir.6472)
Supplement: Multimedia Appendix 1 [file jmir_v19i3e98_app1.pdf]

Appendix 1: Items used for assessing respondents' perceived health outcomes from using the Internet for health purposes

Items used in our survey:

Items used for assessing the respondents perceived health outcomes:

"Do you agree or disagree that seeking health information on the Internet:"

1. Improved your ability to manage your health needs.
2. Enabled you to ask your physician questions resulting from the information you acquired on the Internet.
3. Enabled you to show your physician the information that you retrieved.
4. Increased your sense of power in your encounter with the physician.
5. Improved your understanding of the symptoms, conditions, or treatments in which you were interested.
6. Revised your knowledge of health innovations.
7. Led you to take independent steps (such as seeing a specialist, or changing an exercise regimen or eating habits).
8. Enabled you to think about alternative treatment options.
9. Made you more aware of patients' insurance-related rights.

Responses were expressed on a 5-point scale from 1 (*strongly agree*) to 5 (*strongly disagree*).
