# Supplementary material for: With Some Help From My Network: Supplementing eHealth Literacy With Social Ties
Source: J Med Internet Res. 2017 Mar 30;19(3):e98. doi: 10.2196/jmir.6472 (PMC5391437; doi:10.2196/jmir.6472)
Supplement: Multimedia Appendix 2 [file jmir_v19i3e98_app2.pdf]

Appendix 2: Descriptive statistics of the "ease of obtaining help in performing online activities", "perceived eHealth literacy", "finding others with similar health concerns", and the perceived outcome of Internet use measures.

Item used to measure Ease of obtaining help in performing online activities	Average score for Israeli Jews (SD)	Average score for PCI (SD)
When you need advice or help surfing the Internet, for example help in finding a particular site or service, how easy is it for you to find someone who will help you?	2.04 (2.1) (n=677)	1.45 (2.12) (n=135)

Item used to measure finding others with similar health concerns	Average score for Israeli Jews (SD)	Average score for PCI (SD)
During the past 12 months, how often have you used the Internet for finding others with health issues or concerns similar to the ones you are facing?	1.78 (.85) (n=682)	1.6 (.78) (n=136)

Item used to measure perceived eHealth literacy	Average score for Israeli Jews (SD)	Average score for PCI (SD)
I know how to find helpful health resources on the Internet	1.8 (1.93) (n=680)	1.58 (1.97) (n=136)
I know how to use the Internet to answer my health questions	1.79 (1.93) (n=678)	1.66 (2.05) (n=136)
I know what health resources are available on the Internet	1.8 (1.93) (n=680)	1.58 (1.97) (n=136)
I know where to find helpful health resources on the Internet	1.82 (1.94) (n=679)	1.59 (1.97) (n=136)

I know how to use the health information I find on the Internet to help me	1.69 (1.87) (n=669)	1.54 (1.94) (n=163)
I have the skills I need to evaluate the health resources I find on the Internet	1.69 (1.84) (n=675)	1.46 (1.82) (n=136)
I can tell high quality from low quality health resources on the Internet	1.6 (1.82) (n=677)	1.39 (1.76) (n=136)
I feel confident in using information from the Internet to make health decisions	1.4 (1.63) (n=676)	1.29 (1.66) (n=136)

**Item used to measure perceived health outcomes**	**Average score for Israeli Jews (SD)**	**Average score for PCI (SD)**
Improved your ability to manage your health needs.	1.27 (1.56) (n=672)	1.22 (1.66) (n=136)
Enabled you to ask your physician questions resulting from the information you acquired on the Internet.	1.7 (1.93) (n=672)	1.48 (1.84) (n=136)
Enabled you to show your physician the information that you retrieved.	1.5 (1.77) (n=666)	1.37 (1.77) (n=135)
Increased your sense of power in your encounter with the physician.	1.24 (1.58) (n=667)	1.51 (1.9) (n=136)
Improved your understanding of the symptoms, conditions, or treatments in which you were interested.	1.68 (1.87) (n=671)	1.55 (1.89) (n=136)
Revised your knowledge of health innovations	1.6 (1.82) (n=671)	1.43 (1.8) (n=136)
Led you to take independent steps (such as seeing a specialist, or changing an exercise regimen or	1.51 (1.8) (n=667)	1.4 (1.79) (n=136)

eating habits).<br>Enabled you to think about alternative treatment options.		
Enabled you to think about alternative treatment options.	1.4 (1.71)<br>(n=669)	1.37 (1.77)<br>(n=136)
Made you more aware of patients’ insurance-related rights.	1.38 (1.72)<br>(n=658)	1.36 (1.75)<br>(n=135)
